# Tumor Suppressor Role and Clinical Significance of the *FEV* Gene in Prostate Cancer

**DOI:** 10.1155/2022/8724035

**Published:** 2022-05-02

**Authors:** Yu-Xiang Liang, Ying-Ke Liang, Zhi-Hao Zou, Yang-Jia Zhuo, Jian-Heng Ye, Xue-Jin Zhu, Zhou-Da Cai, Zhuo-Yuan Lin, Ru-Jun Mo, Shu-Lin Wu, Yan-Qiong Zhang, Wei-De Zhong

**Affiliations:** ^1^Department of Urology, Guangdong Key Laboratory of Clinical Molecular Medicine and Diagnostics, Guangzhou First People's Hospital, School of Medicine, South China University of Technology, Guangzhou 510180, China; ^2^Department of Urology, Guangdong Key Laboratory of Clinical Molecular Medicine and Diagnostics, Guangzhou First People's Hospital, Guangzhou Medical University, Guangzhou 510180, China; ^3^Department of Urology, The Second Affiliated Hospital of Guangzhou Medical University, Guangzhou Medical University, Guangzhou 510260, China; ^4^Department of Urology, Affiliated Dongguan Hospital, Southern Medicine University, Dongguan 523059, China; ^5^Departments of Urology and Pathology, Massachusetts General Hospital and Harvard Medical School, Boston, MA 02114, USA; ^6^Institute of Chinese Materia Medical, China Academy of Chinese Medical Sciences, Beijing 100700, China; ^7^Department of Urology, Huadu District People's Hospital, Southern Medical University, Guangzhou 510800, China; ^8^School of Medicine, Jinan University, Guangzhou, Guangdong 510632, China

## Abstract

**Background:**

In our previous research, we developed a 32-gene risk index model that may be utilized as a robust prognostic method for predicting prostate cancer (PCa) recurrence after surgery. Among the 32 genes, the Fifth Ewing Variant (*FEV*) gene was one of the top downregulated genes in relapsed PCa. However, current understanding of the FEV gene and its involvement in PCa is limited.

**Methods:**

FEV mRNA expression was analyzed and correlated to clinical outcomes in PCa patients who underwent prostatectomy at the Massachusetts General Hospital. Specimens from tissue microarray (TMA) including 102 prostate cancer patients were analysis for the expression of FEV. Meanwhile, FEV expression profiles were also assessed in PCa cell lines and in BPH-1 prostate epithelial cells using western blotting and quantitative reverse transcription-PCR (qRT-PCR). Furthermore, we transfected LNCaP and PC-3 cells with either an empty vector or full-length FEV gene and performed in vitro cell functional assays. The part FEV plays in tumor xenograft growth was also assessed in vivo.

**Results:**

Of the 191 patients included in this study base on the DASL dataset, 77 (40.3%) and 24 (13.6%), respectively, developed prostate-specific antigen (PSA) relapse and metastasis postradical prostatectomy. Significant FEV downregulation was observed in PCa patients showing PSA failure and metastasis. The protein expression of FEV was significantly negatively correlated with the Gleason score and pathological stage in prostate cancer tissues. Similarly, FEV expression significantly decreased in all PCa cell lines relative to BPH-1 (all *P* < 0.05). Functional assays revealed that FEV expression markedly inhibited PCa cell growth, migration, and invasion, which in turn significantly repressed the growth of tumor xenografts in vivo.

**Conclusion:**

The results of this study suggest an association between downregulated FEV expression and PSA relapse in PCa patients. In addition, FEV may act as a tumor suppressor in PCa.

## 1. Introduction

Prostate cancer (PCa) is the most frequently diagnosed type of nondermatologic cancer and the second major cause of cancer-associated death in males in the US. In 2018, approximately 174,650 men in the US have been diagnosed with PCa, of which 31,620 have died of this disease [1]. Although early diagnosis allows the opportunity for curative surgery, around 35% of men who have undergone radical prostatectomy have prostate-specific antigen (PSA) recurrence within 10 years after the operation and it is often a result of micrometastatic disease occurring at the time of surgery [2–5]. It has thus become a challenge to identify individuals who are at high risk for relapse as well as those who may benefit from secondary treatment yet can negatively affect their quality of life [6]. Different variables have been employed in classifying patients who are at low, intermediate, and high risk for tumor recurrence after local therapy; these include standard pre and postoperative clinical and pathological variables including tumor stage, serum PSA levels, and Gleason scores [7–9]. However, the clinical features of low- and intermediate-risk patients are similar, and thus, predicting outcomes for these patient groups is difficult. There is thus a need to identify molecular abnormalities that distinguish those at high risk for relapse.

The method of prostate tumor specimen expression profiling via oligonucleotide or cDNA microarray technology has been utilized in the detection of gene expression signatures for PCa prognosis. Various molecular markers have been identified by gene expression profiling, which include AMACR [10], BMI-1 [11], EZH2 [12], PCA3 [13], and TMPRSS2-ERG [14**]**. In our earlier study [15], we employed a microarray-based gene expression profiling technique to identify a 32-gene risk index model that could distinguish PCa patients based on outcomes after surgical treatment. Notably, among the 32 genes selected, the Fifth Ewing Variant (*FEV*) gene showed the most significant downregulated expression in recurrent PCa.

The *FEV* gene is part of the ETS transcription factor family; this gene is located on chromosomal band 2q36 and consists of three exons. The human FEV protein comprises 238 amino acids that have 96**%** sequence similarity to the 237-amino acid murine Pet-1 protein [16]. FEV is specifically expressed in the prostate, small intestine, and serotonin-containing neurons of the brain. To the best of our knowledge, the molecular function of FEV in prostate or PCa has not been examined thus far. Hence, the current study investigated FEV mRNA expression as well as assessed its correlation to PCa patient outcomes after prostatectomy from 1993 to 1995 at the Massachusetts General Hospital. We also assessed FEV expression levels in PCa and prostate epithelial cell lines by quantitative real-time reverse transcription-polymerase chain reaction (qRT-PCR) and western blotting analysis. To further investigate the function of *FEV* in PCa cells, we also established stable FEV-overexpressing PCa cell lines, which were used in in vitro cell proliferation, cell migration and invasion, and cell cycle assays. We also investigated the part FEV plays in the growth of tumor xenografts in vivo using gain-of-function experiments.

## 2. Materials and Methods

### 2.1. Patients and Tissue Samples

This study received approval from the Human Study Ethics Committee of Massachusetts General Hospital (MGH; Boston, MA, USA) and was conducted based on the guidelines described in the Declaration of Helsinki. All specimens were handled and deidentified following the ethical and legal standards. All study participants provided their written informed consent.

A total of 191 tissue samples were collected from patients who had undergone RP surgery as part of their treatment regimen at the Massachusetts General Hospital (MGH; Boston, MA, USA) from September 1993 to September 1995. A review of medical records was performed, and clinical data such as demographic information, presurgery PSA, Gleason score, pT stage, surgical margin status, time to metastasis, time to biochemical recurrence (BCR), and overall survival (Table 1). A single urological pathologist (C.-L.W.) reviewed the Gleason scores using the modified Gleason classification [17].

Survival information was collected from the MGH medical records as well as from social security database inquiries. When a confirmation of death was unavailable, the final confirmed patient follow-up visit during which a PSA test was performed to represent time of overall survival analyses. The median clinical follow-up time to biochemical failure event, no biochemical failure event, death, and no death event were 3.10 (range: 0.06–12.58), 9.39 (range: 0.23–15.81), 11.41 (range: 3.65–15.67), and 13.65 (range: 0.25–16.27), respectively. The exclusion criteria were neoadjuvant or adjuvant therapy prior to BCR and occurrence of lymph node metastasis at RP.

All tissue samples were formalin-fixed and paraffin-embedded. The tumor samples were sliced into 10 × 10-*μ*m-thick tissue sections, and the region of the tissue showing the highest histologic tumor grade was identified by a pathologist (C.-L.W.) for isolation via manual microdissection. The lower limit for tumor tissues that were accepted was 70%. cDNA-mediated annealing, selection, extension, and ligation (DASL) bead microarray assay was conducted at the Mayo Clinic College of Medicine Genotyping Shared Resource (Rochester, MN, USA). The DASL panel was synthesized by Illumina and is available online (http://www.ncbi.nlm.nih.gov/projects/geo; Accession No. GSE44353). The TCGA dataset (https://cancergenome.nih.gov/) was downloaded from the cBioPortal for Cancer Genomics (http://www.cbioportal.org/). We employed the median value of FEV expression levels in PCa tissues in the TCGA dataset and DASL database as cutoff, low expression group was defined when FEV expression was less than the median, while the rest comprised the high expression group.

### 2.2. Cell Culture

Four human PCa cell lines (LNCaP, DU-145, PC-3, and 22Rv1) as well as the immortalized prostate epithelial cell line BPH-1 were purchased from the American Type Culture Collection and cultured in RPMI 1640 or DMEM that was supplemented with 10% fetal bovine serum (Thermo Fisher Scientific, Waltham, MA, USA) and penicillin (50 IU/mL)/streptomycin (50 *μ*g/mL) (Thermo Fisher Scientific, Waltham, MA, USA).

### 2.3. FEV-Overexpressing PCa Cell Model

To generate FEV-overexpressing stable cells, two PCa cell lines LNCaP and PC-3 were transfected with FEV Human cDNA Clone or pCMV6-neo vector following the manufacturer's protocol (Origene, Rockville, MD, USA). Forty-eight hours after transfection, the cells were selected using G418 (1.2 mg/mL) (Cellgro, Manassas, VA, USA). Stable LNCaP and PC-3 cell lines overexpressing FEV were generated and cultured in complete RPMI 1640 with G418 (1.2 mg/mL). FEV overexpression was then confirmed using western blot and real-time RT-PCR.

### 2.4. Generation of In Vivo Xenograft Model

Every animal experiment was conducted following the guidelines of the Institute for Laboratory Animal Research of Guangzhou Medical University, Guangzhou, P.R. China. Twenty BALB/c nude mice (4- to 5-week-old males) were purchased from the Guangdong Medical Laboratory Animal Center. Five mice were kept in each wire-top cage with sawdust bedding that was placed in a clean, isolated room with a controlled temperature of 25°C–26°C, ~50% relative humidity, and illumination at 12 h/day.

### 2.5. In Vivo Tumor Formation

FEV-overexpressing PC-3 cells were collected by trypsinization and resuspended in phosphate-buffered saline (PBS). Subsequently, the cells were introduced into the flanks of the nude mice (5 mice per group) by subcutaneous injection. The PC-3 cells were introduced as a suspension of 2 × 10^6^ cells and an equal volume of Matrigel (Cat No: 356234, BD Biosciences), with a total concentration of 10 mg/mL. Measurement of tumor sizes was performed at 4-day intervals once the tumors were measurable. Tumor volume was calculated as follows: V (mm^3^) = Width^2^ (mm^2^) × Length (mm)/2. The mice were humanely euthanized on day 44. The method of euthanasia was as follows: mice were kept in a small cage containing 100% CO_2_ for one hour until respiratory arrest. The mice were housed and handled following the protocols endorsed by the Institute for Laboratory Animal Research of Guangzhou Medical University. All animal experiments were approved and were in accordance with the Institute for Laboratory Animal Research of Guangzhou Medical University's guidelines (Guangzhou, PR China).

### 2.6. Western Blot

FEV protein expression levels in PCa cells were assessed with western blotting analysis as previously detailed [18, 19]. The antibodies utilized in this research were as follows: FEV (Abnova, Cat. #H00054738-A01) and *β*-actin (Sigma, Product# A5316). All experiments were conducted thrice.

### 2.7. qRT-PCR

FEV mRNA expression levels in PCa cells and clinical PCa tissues were assessed by qRT-PCR analysis as detailed in our prior research [18, 19]. The primer sequences utilized in this research were as follows: FEV (F′: CAACATGTACCTGCCAGATCCC, R′: GGTCCGTGAGCTTGAACTCG); GAPDH (F′: AGCGAGCATCCCCCAAAGTT, R′: GGGCACGAAGGCTCATCATT).

### 2.8. Cell Viability Assay

Cell viability was determined utilizing the CCK-8 assay as detailed in our prior research [18, 19].

### 2.9. Cell Invasion and Migration Assays

Cell invasion and migration were evaluated using the Transwell and the scratch wound-healing motility assays as detailed in our prior research [18, 19].

### 2.10. Apoptosis Detection

Cell apoptosis was assessed using an APC-conjugated Annexin V (Annexin V-APC) kit (Cat No: 550474, BD Biosciences, USA) and 7-aminoactinomycin D (7-AAD) (Cat No: AP104-60-AAD, Multisciences, China) as described in our previous studies [18, 19].

### 2.11. Cell Cycle Analysis

The impacts of FEV on cell cycle progression were assessed using fluorescence-activated cell sorter (FACS) analysis and the Cell Cycle Analysis Kit (Beyotime Institution of Biotechnology, Shanghai, China) following the company's instructions [20].

### 2.12. Immunohistochemistry (IHC)

Human PCa tissue microarray was purchased from the Shanghai Outdo Biotech Company (Shanghai, China). The IHC assay was conducted to explore the cellular distribution and protein expression of FEV in clinical prostate cancer patients and benign prostate specimens. The specimens were incubated with anti-FEV (1 : 500, homemade, Immunogen: CPDPVGDGLFKDGKNPS). The detail for IHC assay was as described in our previous studies [18, 19].

### 2.13. Time-Dependent ROC Curve Analysis

The FPKM data of TCGA RNA-seq datasets for PCa were acquired from the UCSC Xena browser (https://xenabrowser.net/). We changed the type of gene expression profiles from log2(FPKM+1) to log2(TPM+1) to obtain a more precise data of differentially expressed genes (DEGs). An ROC curve was generated under the nonparametric distribution assumption for FEV by plotting sensitivity vs (1-specificity). The “sklearn” package in python was applied for constructing the diagnostic efficacy of FEV in PCa.

### 2.14. Statistical Analysis

Statistical analysis was performed with SPSS 13 (IBM, Chicago, IL, USA) and SAS 9.1 (SAS Institute, Cary, NC, USA). All experiments were performed thrice, and the data were expressed as the mean ± SD. Statistical analysis of the results of western blotting was conducted using the Wilcoxon signed-rank test. Statistical analysis was independently conducted by two biostatisticians using Fisher's exact test for any 2 × 2 contingency tables and Pearson's *χ*^2^ test for non-2 × 2 tables. The Kaplan-Meier method was employed for the survival analysis, and Cox regression analysis was utilized for the univariate and multivariate analyses. Differences with probability level of *P* < 0.05 were deemed to be statistically significant.

## 3. Results

### 3.1. FEV Downregulation in PCa Tissues and Cells

To characterize genes that have been implicated with biochemical failure of PCa postradical prostatectomy, we employed a statistical technique known as random forest classification [15]. Here, the gene at the top of the list of downregulated genes observed in relapsed PCa was the *FEV* gene (*P* = 0.004) [15].

**Figure 1:**
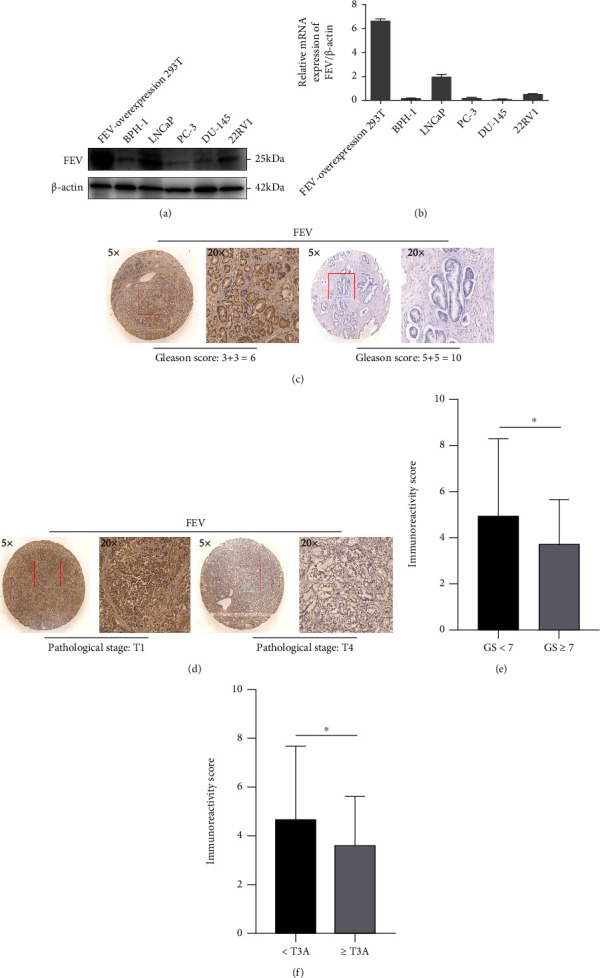
FEV protein expression levels (a) and mRNA expression levels (b) in prostate and prostate cancer cell lines. FEV-overexpression 293 cell was used as the positive control. Representative photographs of FEV protein immunohistochemistry staining in TMA. FEV protein expression level in low Gleason score (GS) (c and e) and low pathological stage (d and f) was significantly higher than that in high GS and high pathological stage.

To validate the *FEV* expression pattern in PCa, western blot analysis of four human PCa cell lines (LNCaP, DU-145, PC-3, and 22Rv1) and a nonmalignant human prostate epithelial cell line (BPH-1) was performed. FEV-overexpressing 293 T cell lysates (Abnova, Taipei, Taiwan) were used as the positive control in western blotting and qRT-PCR. All PCa cell lines showed weak FEV protein expression compared to BPH-1 cells, which showed strong FEV protein expression (all *P* < 0.05, Figure 1).

Next, we explored the correlation between the mRNA expression of FEV and clinicopathological characteristics of PCa patients in the DASL database and TCGA database. Clinical correlation analysis revealed that high mRNA expression of FEV was significantly associated with the Gleason Score, pathological stages, surgical margin, metastasis, and PSA failure (all *P* < 0.05, Table 1). Furthermore, we detected the protein expression of FEV in human PCa patients' samples. The IHC assay was employed to investigate the expression patterns of FEV in human prostate cancer specimen. As shown in Figure 1 and Table 2, high protein expression of FEV was significantly correlated with low Gleason score (*P* < 0.05) and low pathological stage (*P* < 0.05).

### 3.2. FEV Downregulation Is Correlated with Shorter BCR-Free Survival of PCa Patients

**Figure 2:**
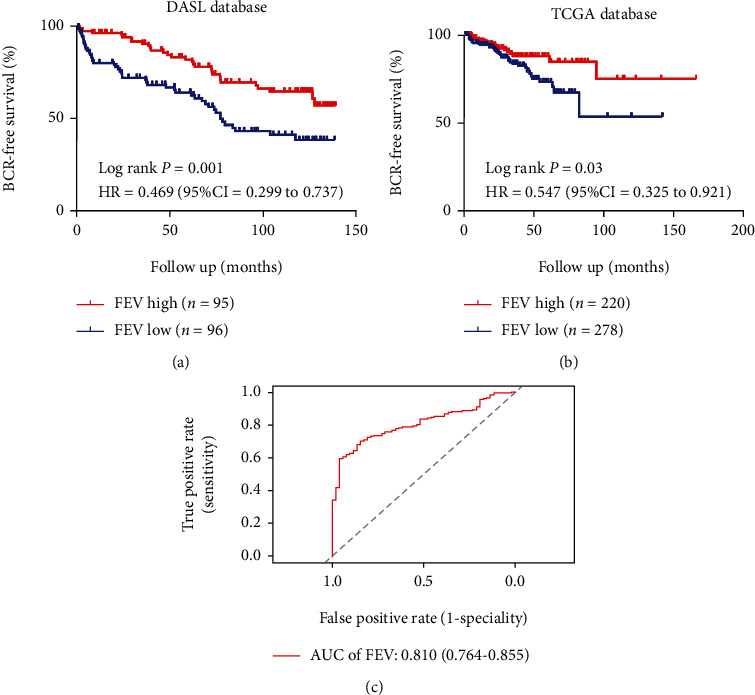
FEV downregulation is associated with shorter biochemical recurrence- (BCR-) free survival in PCa patients. The Kaplan-Meier analyses were performed to assess the prognostic value of FEV in relation to BCR-free survival according to its expression in our cohort (a) and the TCGA cohort (b). ROC curves testing the diagnostic value of FEV (c) in TCGA cohort.

To determine the prognostic value of FEV expression in PCa, we used the Kaplan-Meier method to assess the correlation between FEV expression and BCR-free survival of PCa patients in our cohort DASL and the TCGA dataset. Pairwise comparisons revealed a significant difference in BCR-free survival across patients who exhibited high or low FEV expression levels (both *P* < 0.05, Figures 2(a) and 2(b)). Multivariate analysis revealed that FEV downregulation may be potentially utilized as an independent predictor for a shorter BCR-free survival period (Table 3).

To identify the diagnostic efficacy of FEV in PCa, we performed time-dependent ROC curve analysis. The AUC values for predicting the diagnostic efficacy of FEV in PCa tissues from benign tissues was 0.810 in the TCGA cohort, which revealed that FEV could distinguish PCa tissues from benign tissues (Figure 2(C)).

### 3.3. FEV Overexpression Disrupts PCa Cell Proliferation, Migration, Invasion, and Cell Cycle Whereas Promotes Apoptosis In Vitro

**Figure 3:**
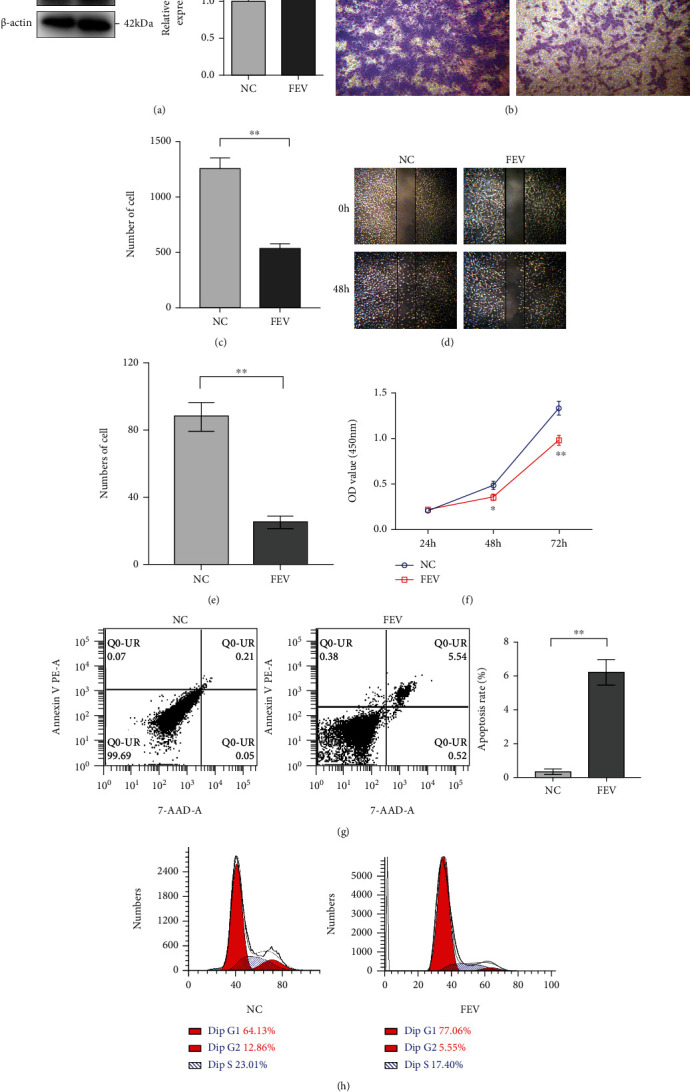
FEV overexpression disrupts LNCaP cell proliferation, invasion, and migration yet promotes apoptosis in vitro. (a) Enforced expression of FEV in the FEV-stably transfected LNCaP cells verified by western blot and qRT-PCR. (b) Transwell analysis revealed that FEV overexpression suppresses the invasive ability of LNCaP cells. The results of the statistical analysis of three independent experiments are shown in panel (c). (d) Wound healing assays showed that FEV upregulation inhibits the migration of LNCaP cells. The results of statistical analysis of three independent experiments are shown in panel (e). (f) CCK-8 assays revealed that FEV overexpression disrupts the proliferation of LNCaP cells. (g) Enforced expression of FEV-induced apoptosis of LNCaP cells. The results of statistical analysis of three independent experiments are shown in the panel. (h) Enforced expression of FEV inhibits the cell cycle of LNCaP cells. Data were presented as the mean ± SEM. ^∗^*P* < 0.05, ^∗∗^*P* < 0.01 relative to the negative control.

**Figure 4:**
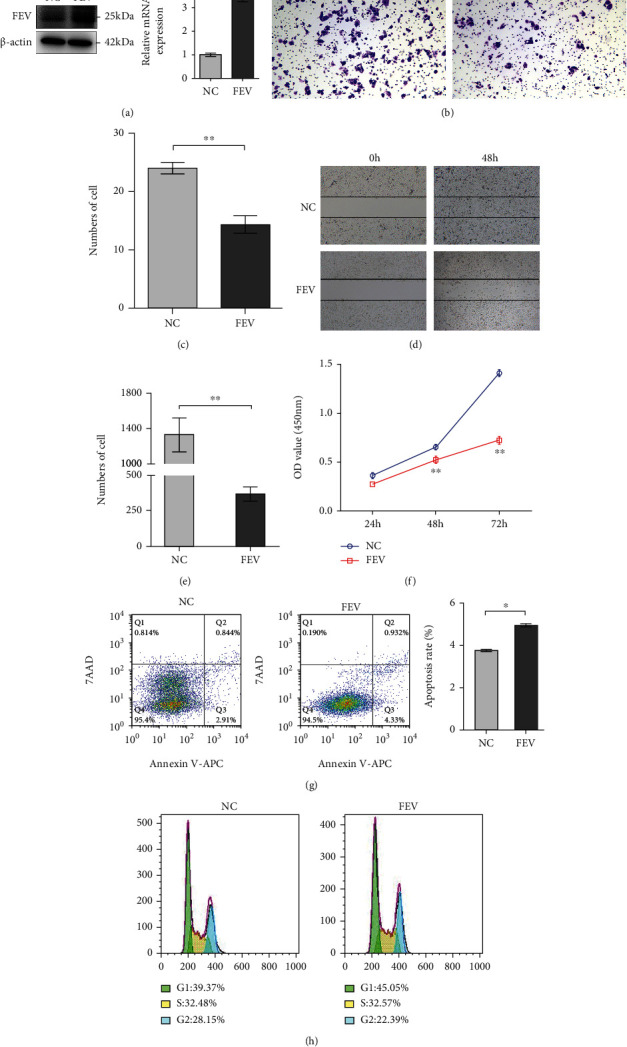
FEV overexpression disrupts PC-3 cell proliferation, invasion, migration, and cell cycle yet promotes apoptosis in vitro. (a) Enforced expression of FEV in the FEV-stably transfected PC-3 cells verified by western blot and qRT-PCR. (b) Transwell analysis revealed that FEV overexpression disrupted the invasive ability of PC-3 cells. The results of statistical analysis of three independent experiments are shown in panel (c). (d) Wound healing assays showed that FEV upregulation disrupts the migration of PC-3 cells. The results of statistical analysis of three independent experiments are shown in panel (e). (f) CCK-8 assays demonstrated that FEV overexpression impaired proliferative activity of PC-3 cells. (g) Enforced expression of FEV-induced apoptosis of PC-3 cells. (h) Enforced expression of FEV suppressed the cell cycle of PC-3 cells. The data are presented as the mean ± SEM. ^∗^*P* < 0.05, ^∗∗^*P* < 0.01 relative to the negative control.

To elucidate the tumor-suppressive role of FEV in PCa, we initially established a stable cell line that overexpressed FEV after lentivector transduction. Western blot and qRT-PCR analysis verified that the FEV-overexpressing LNCaP cells were successfully established (Figure 3(a)). Transwell assays clearly showed that enforced FEV expression significantly decreased the invasive activities of LNCaP cells relative to the control cells (Figures 3(b) and 3(c)). Wound-healing assays showed that FEV upregulation markedly decreased the migratory abilities of LNCaP cells (Figures 3(d) and 3(e)). CCK-8 assays revealed a significantly lower rate of proliferation of FEV-overexpressing LNCaP cells in contrast to the control vector-transfected cells (Figure 3(f)). Further, we also determined that induced FEV expression disrupted the cell cycle of LNCaP cells (Figure 3(h)). However, we observed significantly higher apoptotic rates in FEV-transfected LNCaP cells in contrast to control cells (Figure 3(g)). The above results based on LNCaP cells were in line with those of PC-3 cells shown in Figure 4.

### 3.4. FEV Overexpression Disrupts Tumor Xenograft Growth In Vivo

**Figure 5:**
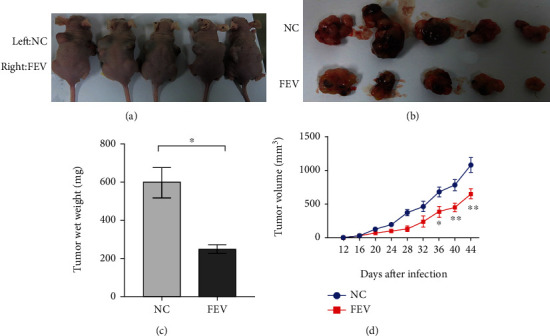
FEV overexpression suppresses tumor xenografts growth in vivo. (a and b) Lentivector-mediated overexpression of FEV in PC-3 cells completely blocked subcutaneous tumor regeneration. Tumor growth was monitored for 44 days (PC-3) after tumor cell injection. (c) Tumor wet weights in FEV overexpression and negative control groups were shown. (d) A tumor growth curve is shown. PC-3 (*n* = 5) cells transfected with FEV-expressing lentivectors or the mock control were administered to nude mice by subcutaneous injection. The tumor sizes were measured at four-day intervals as soon as the tumors were measurable. ^∗^*P* < 0.05 and ^∗∗^*P* < 0.01 using the Student's *t*-test. The data are presented as the mean ± SEM.

To investigate the biological functions of FEV in vivo, FEV-expressing PC-3 cells were introduced into the flank of each male nude mouse via subcutaneous injection. At the same time, we subcutaneously injected the vector control PCa cell lines (NC) into the other flank of the same mice. The FEV-expressing PC-3 cells resulted in the formation of significantly smaller tumor nodules (Figure 5(a)) as well as remarkably slowed the growth of tumor xenografts relative to the controls (Figure 5(b)). Statistically, we further found that the PCa cells that permanently overexpressed FEV could efficiently suppress tumor wet weight and tumor volume in contrast to the controls (both *P* < 0.05, Figures 5(c) and 5(d)).

## 4. Discussion

The *FEV* gene was initially cloned as a chromosomal translocation in a human Ewing tumor in a pediatric case; a portion of this gene was fused to a portion of the sequence that encoded the RNA-binding protein EWS [21]. Although several ETS genes are continuously expressed in all tissues, FEV expression occurs specifically in the small intestine [21], prostate [21], and serotonin- (5-HT-) containing neurons of the brain [22]. No human fetal tissues can express FEV. In serotonergic neurons, FEV directly binds to multiple FEV elements in the human 5-HT1A receptor promoter to improve its transcriptional activity. Alterations in the expression of the human FEV gene influence CNS serotonin neuron gene expression levels and maternal nurturing. In the small intestine, FEV expression occurs in serotonin-producing cells in normal tissues and carcinoid tumors. As far as we know, this is the initial study to elucidate the molecular functions of FEV in PCa.

Extensive evidence has shown that PSA screening worldwide has resulted in overdiagnosis of PCa, which in turn has led to an overtreatment of patients with indolent disease [23]. Hence, it is essential to determine novel and more efficient biomarkers that can distinguish indolent and aggressive PCa so that patients at low risk of progression can avoid unnecessary treatments. Herein, on the basis of the gene expression profiles in relapse and nonrelapse PCa obtained in our previous study [15], as well as the validation of the results of qRT-PCR and western blot analyses, we have verified the downregulation of the FEV gene and protein in PCa tissues and cell lines. Meanwhile, we also investigate that FEV expression was significantly associated with the Gleason score, pathological stage, surgical margin, and PSA failure and metastasis, indicating that FEV expression was negatively correlated with PCa progression. Furthermore, we have also established a significant association between FEV downregulation and shorter BCR-free PCa patient survival in both our cohort as well as in the TCGA dataset, thereby prompting us to elucidate the contribution of FEV to malignant phenotypes of PCa in vitro systems and in vivo models. ROC analyses reveal that FEV expression provided significant result between prostate cancer tissues and benign tissues, indicating that FEV might be the potential diagnosis marker.

In our in vitro research, we discovered that PCa cell lines demonstrated weak expression of FEV compared to BPH-1 cells, indicating that FEV expression levels may be correlated to PCa malignancy. The regulatory mechanism of FEV mRNA expression in normal and cancerous prostate epithelial cells remains unclear. In the central nervous system, FEV expression is controlled by a serotonergic transcriptional cascade that consists of the homeodomain factor *Nkx2.2* [24], proneural factor *Mash1* [25], the zinc finger factor *Gata-2* in postmitotic precursors [26], and the forkhead box factor *Foxa2* [27] in the ventral hindbrain progenitors, and gene fusion with ETS members caused by chromosomal translocation is frequent in human cancers. About 85% of Ewing sarcoma cases involve EWS:FLI-1 gene fusions that are generated by the balanced chromosomal translocation t(11; 22)(q24; q12) [28, 29]. A fusion between ERG and an androgen receptor-regulated gene promoter of TMPRSS2 has been observed in about half of PCa cases, and this results in an abnormal androgen-regulated ERG expression [30, 31]. It is unknown whether chromosomal translocation of FEV occurs in PCa and whether this could affect FEV expression. Our data showed the possible posttranscriptional regulation of FEV expression.

Our in vitro and in vivo PCa models have shown that FEV suppresses tumor growth and invasiveness. These findings are assumed to be due to FEV-mediated transcriptional repression. FEV has been initially suggested as a transcriptional repressor of the ETS family. All ETS family members harbor a highly conserved DNA-binding domain (ETS domain) that is a winged 85-amino-acid helix-turn-helix structure that can bind to DNA sites carrying a central 5′-GGAA/T-3′ motif [32]. Because the ETS domain is highly similar in sequence to Fli-1 and ERG, FEV apparently belongs to the FLI-1/ERG subgroup [21]. However, in addition to the DNA-binding domain, FEV possesses distinctive characteristics. FLI-1 and ERG contain large N-terminal domains that are involved in transcription regulation. Specifically, these domains can promote transcription activation of reporter genes that contain ETS-binding sites [33–36]. The lack of an N-terminal domain in FEV implies that this protein does not share similar transcriptional activation properties as FLI-1 and ERG. Further, numerous alanine residues occur in the C-terminal part of FEV, a property that has been noted in other transcription repressors [37], which indicates that FEV is a repressor that belongs to the FLI-1/ERG subfamily. Maurer et al. have shown that FEV inhibits ETS-dependent reporter genes, which involves both the ETS and the alanine-rich C-terminal domains [38].

The target genes of FEV remain unclear. The similarity of DNA-binding domains between FEV and FLI-1/ERG implies that FEV may change the transcription profile of similar target genes other than FLI-1 or ERG. In Ewing tumors, inactivation of EWS:FLI-1 fusion using RNA interference results in a complete arrest of growth as well as dramatic increase in apoptosis rates. Gene profiling of Ewing cells carrying an inactivated EWS:FLI-1 fusion gene has identified two highly significant functional clusters. The first group includes various genes that are involved in signal transduction, specifically receptor binding. This particular group consists of secreted regulators of the epidermal growth factor, Wnt, and insulin-like growth factor 1 (IGF-1) pathways, and intracellular inhibitors of the MAPK and STAT pathways. The other functional cluster includes various molecules that are involved in the adhesion, formation, and remodeling of the extracellular matrix (ECM) [39]. Several studies have revealed that TMPRSS2: ERG gene fusion induces PCa in both mouse and humans with concurrent loss of PTEN [40]. Transcriptional profiling of ERG knockdown in the TMPPRSS2: ERG-positive PCa cell line VCaP (VCaP-siERG) has shown 265 and 291 features that are over and underexpressed, respectively, in VCaP-siERG relative to VCaP treated with nontargeting siRNA [41]. However, whether FEV targets the transcription of similar genes than FLI-1 or ERG requires further investigation.

In typical regulation, ETS expression is closely controlled to regulate various biological processes such as cell proliferation, metastasis, apoptosis, angiogenesis, and transformation. In cancer, the abnormal expression of oncogenic transcription factors (e.g., FLI-1 and ERG) as well as negative regulators of carcinogenesis (e.g., FEV) in the ETS family induces the upregulation of genes that are known to promote cancer and the downregulation of genes that subdue cancer. Thus, therapeutic targeting of ETS factor function may be achieved by reestablishing typical ETS regulatory networks via activation of suppressive ETS factor expression that leads to the inhibition of oncogenic effects. Our study suggests that FEV is a candidate tumor suppressor with decreased or disrupted protein or mRNA expression in PCa. Reexpression of FEV results in the inhibition of cell growth, migration, and invasion. Elucidating the mechanism(s) that mediate FEV downregulation in aggressive PCa may facilitate in the identification of signaling pathways and/or coregulatory factors that could be modulated using therapeutic means to reactivate FEV expression in cancer cells.

In summary, we analyzed tissues from patients who underwent prostatectomy due to clinically localized PCa. We observed that reduced FEV expression is potentially associated PSA relapse. Further, our in vitro and in vivo studies revealed that FEV suppresses PCa cell growth and invasiveness. These results from this study suggest that FEV possibly functions as a tumor suppressor in PCa.

## Figures and Tables

**Table 1:** Correlation between FEV mRNA expression and various clinicopathological characteristics of PCa patients.

Clinical features	FEV with median subtraction in DASL dataset (*N* = 191)			FEV in TCGA dataset (*N* = 498)		
	Cases	Mean ± S.D.	*P*	Cases	Mean ± S.D.	*P*
*Age (years)*
<66	127	−0.12 ± 0.75	0.248	354	7.84 ± 1.98	0.082
≥66	64	0.15 ± 0.77		142	7.50 ± 1.90	
*Serum PSA levels (ng/mL)*
<4	19	−0.30 ± 0.86	0.090	52	8.02 ± 1.87	0.280
≥4	103	0.01 ± 0.68		422	7.71 ± 1.93	
*Gleason score*
<7	69	0.14 ± 0.69	**<0.001**	44	8.50 ± 1.58	**<0.001**
=7	97	−0.04 ± 0.64		247	8.28 ± 1.33	
>7	25	−0.822 ± 0.94		205	6.94 ± 2.36	
*Pathological stage*
T2 < T3A	144	0.00 ± 0.74	**0.015**	177	8.02 ± 1.93	**0.026**
T3 ≥ T3A	47	-0.31 ± 0.79		128	7.60 ± 2.05	
*Surgical margin*						
Negative	114	−0.03 ± 0.73	0.358	312	8.02 ± 1.69	**<0.001**
Positive	77	−0.14 ± 0.80		151	7.22 ± 2.26	
*PSA failure*
Negative	114	0.57 ± 0.67	**0.003**	370	7.85 ± 1.87	**0.021**
Positive	77	−0.27 ± 0.84		59	6.99 ± 2.68	
*Metastasis*
No	153	−0.03 ± 0.74	**0.037**	415	8.00 ± 1.67	**<0.001**
Yes	24	-0.38 ± 0.73		82	6.44 ± 2.66	
*OS*
Alive	158	−0.06 ± 0.77	0.169	486	7.75 ± 1.93	0.708
Died	19	−0.27 ± 0.59		10	7.51 ± 3.26	

**Table 2:** Associations of FEV expression with clinicopathological parameters of patients with PCa.

Clinical features	IRS of FEV protein in TMA (*N* = 102)		
	Cases	Mean ± S.D.	*P*
*Age (years)*
<66	19	6.00 ± 2.64	0.114
≥66	83	5.11 ± 3.38	
*Serum PSA levels (ng/ml)*
<4	—	—	—
≥4	—	—	
*Gleason score*
<7	57	4.95 ± 3.31	**0.023**
≥7	45	3.75 ± 1.89	
*Pathological stage*
<T3A	56	4.71 ± 2.94	**0.015**
≥T3A	46	3.63 ± 2.61	
*PSA failure*
Negative	—	—	—
Positive	—	—	
*Metastasis*
No	—	—	—
Yes	—	—	
*Overall survival*
Alive	—	—	—
Died	—	—	

**Table 3:** Prognostic value of FEV expression in overall survival using a Cox proportional hazards model.

Parameters	Univariate analysis		Multivariate analysis	
	HR (95%CI)	*P*	HR (95%CI)	*P*
DASL dataset (*N* = 191)				
Age (<66 vs. ≥66)	1.265 (0.794-2.015)	0.323		
Preoperative PSA (<4 vs. ≥4)	1.954 (0.776-4.921)	0.155		
Pathological tumor stage (<T3A vs. ≥T3A)	2.336 (1.479-3.785)	**<0.001**	1.344 (0.817-2.210)	0.244
Gleason score (<7 vs. =7 vs. ≥7)	2.978 (2.114-4.195)	**<0.001**	2.539 (1.764-3.655)	**<0.001**
FEV (low *vs.* high)	0.466 (0.294-0.738)	**0.001**	0.606 (0.817-2.210)	**0.038**
TCGA dataset (*N* = 498)				
Age (<66 vs. ≥66)	1.208 (0.676-2.157	0.524		
Pathological tumor stage (<T3A vs. ≥T3A)	3.362 (1.680-6.726)	**0.001**	2.698 (1.340-5.431)	**0.005**
Gleason score (<7 *vs.* =7 vs. ≥7)	3.440 (2.018-5.863)	**<0.001**	3.025 (1.709-5.354)	**<0.001**
FEV (low vs. high)	0.616 (0.362-1.050)	0.075	0.811 (0.456-1.442)	0.475

## Data Availability

The DASL panel was synthesized by Illumina and is available online (http://www.ncbi.nlm.nih.gov/projects/geo; Accession No. GSE44353). The TCGA dataset (https://cancergenome.nih.gov/) was downloaded from the cBioPortal for Cancer Genomics (http://www.cbioportal.org/).
